# Supplementary material for: The Different Clinicopathological Features of Remnant Gastric Cancer Depending on Initial Disease of Partial Gastrectomy
Source: Cancers (Basel). 2020 Oct 2;12(10):2847. doi: 10.3390/cancers12102847 (PMC7601166; doi:10.3390/cancers12102847)
Supplement: Supplementary file 1 [file cancers-12-02847-s001.pdf]

Supplementary Materials

The Different Clinicopathological Features of Remnant Gastric Cancer Depending on Initial Disease of Partial Gastrectomy

Won Ho Han, Bang Wool Eom, Hong Man Yoon, Young-Woo Kim, Myeong-Cherl Kook and Keun Won Ryu

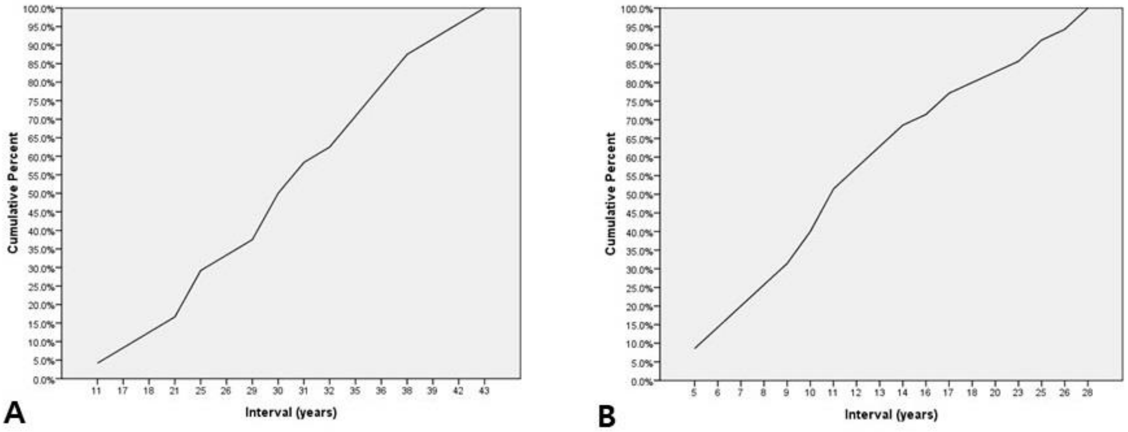

Figure S1. Cumulative incidence of remnant gastric cancer. (**A**) Benign group (**B**)Malignant group.

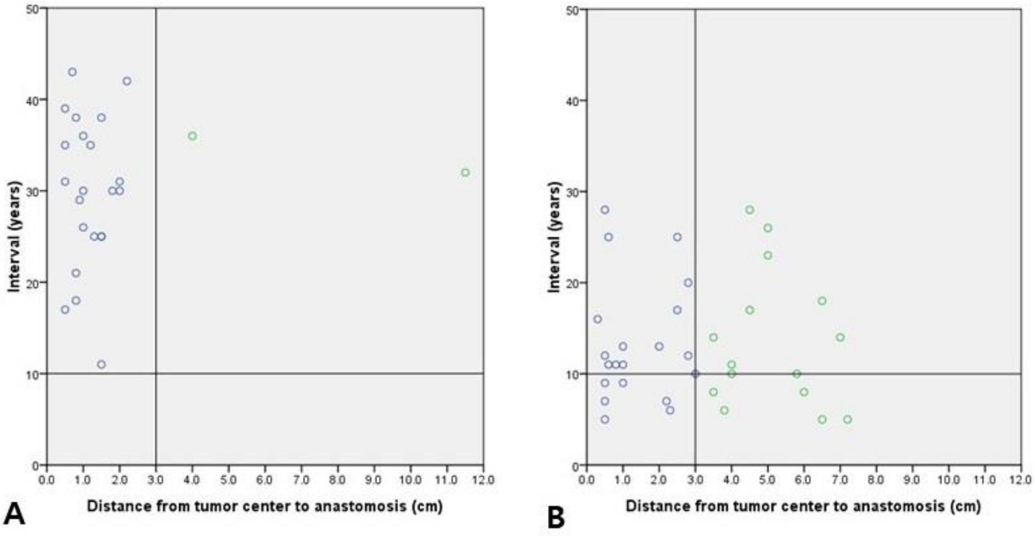

Figure S2. Scatter plot of remnant gastric cancer according to interval and distance from anastomosis site. (**A**) Benign group (**B**)Malignant group.

© 2020 by the authors. Licensee MDPI, Basel, Switzerland. This article is an open access article distributed under the terms and conditions of the Creative Commons Attribution (CC BY) license (http://creativecommons.org/licenses/by/4.0/).
